# MicroRNA and liver cancer

**DOI:** 10.20517/cdr.2019.110

**Published:** 2020-04-17

**Authors:** Masaya Onishi, Takahiro Ochiya, Yasuhito Tanaka

**Affiliations:** ^1^Department of Virology & Liver Unit, Nagoya City University Graduate School of Medical Sciences, Nagoya 467-8601, Japan.; ^2^Department of Molecular and Cellular Medicine, Institute of Medical Science, Tokyo Medical University, Tokyo 160-8402, Japan.

**Keywords:** MicroRNA, hepatocellular carcinoma, diagnosis, extracellular vesicles

## Abstract

Hepatocellular carcinoma (HCC) is a major cause of cancer-related deaths worldwide. HCC is characterized by a poor prognosis and an ever increasing number of scientific studies aim to find new diagnostic, prognostic, and therapeutic targets. MicroRNAs (miRNAs), small non-coding RNAs that regulate the gene expression in many processes, have been shown to play a crucial role in regulating hepatocellular carcinoma. miRNAs may act as oncogenic miRNAs and tumor suppressor miRNAs and regulate cancer cell proliferation, invasion, and metastasis by being differently upregulated or downregulated and targeting the genes related with carcinogenesis. miRNAs secreted from cancer cells are found circulating in the blood, presenting an opportunity for their use as disease-related biomarkers. Moreover, extracellular vesicle-derived miRNAs are known to reflect the cell of origin and function and may provide effective biomarkers for predicting diagnosis and prognosis and new therapeutic target in HCC. In this article, we describe the most recent findings regarding the molecular mechanisms and gene regulation of microRNA in HCC, as well as their application in diagnosis/prognosis and treatment.

## Introduction

Hepatocellular carcinoma (HCC) is considered the second leading cause of cancer-related death worldwide^[1]^. Although several treatment options, such as surgical resection, liver transplantation, percutaneous ablation, and administration of anticancer drugs such as solafenib, are available, a high recurrence rate and unsatisfactory survival rate are major obstacles to the treatment of HCC^[2]^. Because chronic infection by hepatitis viruses, especially hepatitis C virus (HCV), is a main cause of HCC, the application of revolutionary, highly effective direct-acting antiviral agents against HCV, which have recently become available, is a promising approach to reducing future HCC incidence. However, the considerable risk of HCC development even after eradication of HCV remains a challenge^[3]^. In addition, hepatitis B virus (HBV)-related as well as alcohol/non-alcoholic liver disease-related HCC is also an important public health burden. Although extensive efforts have identified several driver genes frequently mutated in HCC^[4,5]^, potentially curative treatments are limited. New drugs (i.e., lenvatinib, regorafenib, cabozantinib, and ramucirumab) have been shown to improve the clinical outcomes of patients with advanced HCC but improvements in terms of survival are modest.

MicroRNAs (miRNAs) are small, non-coding RNAs of approximately 21-23 nucleotides and are broadly conserved in eukaryotes, including plants and animals. miRNAs downregulate the expression of their target genes post-transcriptionally by complementary base pairing with the 3’-untranslated region of messenger RNAs (mRNAs) and, in doing so, control various fundamental biological processes^[6]^. In the last decade, pivotal roles of miRNAs in the development and progression of cancer have become widely been accepted^[7]^. Extensive efforts have shown that the expression profiles of miRNAs are frequently altered in HCC, and miRNAs have been shown to contribute to the development and progression of HCC. In this review, we summarize recent progress in the field of HCC-related miRNAs, application of miRNAs to the diagnosis of HCC, and their therapeutic potential for the treatment of HCC.

## miRNA biogenesis and function

miRNAs are encoded in the genome either as independent gene segments or within the introns of protein-coding genes. miRNA-encoding genes are first transcribed by RNA polymerase II, generating long transcripts called “primary miRNAs” that contain several stem-loop secondary structures^[8]^. After capping at the 5’ end and polyadenylation at the 3’ end, primary miRNAs are processed in the nucleus by the endonuclease Drosha and cleaved into precursor miRNAs, which comprise a mature miRNA sequence and a complementary sequence linked by a short loop region^[9]^. After being transported from the nucleus to the cytoplasm by exprotin-5, the precursor miRNA stem-loop is further processed by RNase III endonuclease Dicer to produce an RNA duplex. The RNA duplex is incorporated into the RNA-induced silencing complex (RISC), which carries out miRNA-mediated post transcriptional regulation of the target genes. Basically, only one strand of the RNA duplex is utilized as a mature miRNA and the opposite complementary strand (miRNA*) is removed from the RISC complex^[10]^. A 6-7 nt sequence complementary to the 5’ end of each miRNA is present in the 3’ untranslated region or, in some cases, other regions of the target mRNAs. The Cyclin D1 (CCND1), Mesenchymal-epithelial transition factor (cMET) and CDK6 suppresses translation by binding to the target mRNA. Because each miRNA can recognize hundreds of target genes and each protein coding gene is controlled by several miRNAs, miRNAs can broadly control complex gene regulatory networks. In addition, recent studies have suggested that some miRNAs contribute to the stability and robustness of gene regulatory networks, which are influenced by environmental or intrinsic fluctuations, such as temperature and genetic/epigenetic variations^[11]^. Collectively, miRNAs serve as fine-tuners for the precise control of various cellular responses.

## Role of miRNA in HCC

In this paper, we introduce the impact of miRNAs on HCC growth, progression, and tumor microenvironment. Several large-scale analyses of miRNA expression profile for HCC provided the key miRNAs. miRNA has many putative miRNA recognition sites, and there are many target genes predicted by computational tools. It is difficult to fully understand their functions, but recent studies have gradually elucidated the role of miRNAs in HCC. Moreover, recent evidence suggests that the epigenetic mechanisms of miRNA expression provide a potential therapeutic target^[11]^. Research of not only the expression but also the methylation of miRNAs may be important to better understand the relationship between miRNA and HCC.

### miRNA expression profiles in HCC

Several studies have been conducted to search for biomarkers or therapeutic agents by investigating the differential expression of miRNAs between hepatocellular carcinoma tissue and the corresponding non-tumor liver tissue.

Andrés-León *et al*.^[12]^ analyzed miRNAs in The Cancer Genome Atlas (TCGA) data for HCC and other tumor types. In 2018, a study integrated miRNA expression database (TCGA, GSE31384^[13]^, and GSE6857^[14]^) and compared their expression in liver cancer and adjacent normal samples to investigate the difference of miRNAs expression associated with carcinogenesis^[15]^. They found that hsa-miR-149, hsa-miR-139, hsa-miR-3677, hsa-miR-550a, and hsa-miR-212 were significantly correlated with overall survival of HCC patients. Li *et al*.^[16]^ also undertook the integrated analysis of a number of miRNA profiling studies^[17-34]^ in HCC samples to detect differentially expressed miRNAs. As the result of analysis, miR-221/222, miR-195, and miR-199a were consistently differentially expressed in multiple independent studies.

Although no overlapping expression patterns of some miRNAs could be found in the existing literature and the currently available data, the miRNAs reported most consistently as dysregulated, namely hsa-miR-18, hsa-miR-21, hsa-miR-106, hsa-miR-221/222, hsa-miR-224, hsa-miR-99, hsa-miR-195, and hsa-miR-199, play a role in regulating the hallmarks of HCC [Table 1].

**Table 1:** Summary of consistently reported up-/downregulated microRNAs in profiling studies

Upregulated in HCC tissues	Ref.
hsa-miR-18a-3p	[12,16,17]
hsa-miR-21-5p	[12,15-19]
hsa-miR-25-3p	[15,19]
hsa-miR-93-5p	[12,18,19]
hsa-miR-96-5p	[12,15]
hsa-miR-106b-5p	[15,17,19]
hsa-miR-151-3p	[17,19]
hsa-miR-151a-5p	[12,17]
hsa-miR-181b-5p	[12,15]
hsa-miR-182-5p	[12,15]
hsa-miR-183-3p	[12,15]
hsa-miR-183-5p	[12,15]
hsa-miR-210	[16,17]
hsa-miR-221	[12,15-17]
hsa-miR-222	[12,15-18]
hsa-miR-224-5p	[12,16-18]
hsa-miR-301b	[12,15]
Downregulated in HCC tissues	
hsa-let-7c-5p	[12,19]
hsa-miR-99a-5p	[16,17,19]
hsa-miR-125b	[16,17]
hsa-miR-130a-3p	[12,18]
hsa-miR-139-3p	[12,15]
hsa-miR-139-5p	[12,15,19]
hsa-miR-142-5p	[12,17]
hsa-miR-145	[12,15,18]
hsa-miR-195	[12,15-18]
hsa-miR-199a-3p	[15,17-19]
hsa-miR-199a-5p	[16,18,19]
hsa-miR-214-3p	[12,15,18]
hsa-miR-326	[12,15]
hsa-miR-424-3p	[12,15]
hsa-miR-424-5p	[12,15]

HCC: hepatocellular carcinoma

### miRNA as tumor suppressors

A tumor suppressor gene is defined as a gene which encodes proteins that inhibit tumorigenesis. Loss or reduction of function in tumor suppressor genes plays an oncogenic role, leading to the development of many tumors. The product of the tumor suppressor genes plays a regulatory role of cell proliferation, cell differentiation and DNA repair. Notably, as shown in Table 1, several miRNAs are significantly downregulated in tumor tissues and may be the characteristics of tumor suppressor genes.

Recent studies have revealed that miR-199 inhibited HCC proliferation and metastasis. miR-199a/b-3p can suppress HCC growth *in vitro* and *in vivo* by inhibiting inhibiting p21-Activated kinase 4 (PAK4), which is known to activate Raf/MEK/ERK pathway^[35]^. Moreover, in another study, miR-199a-5p was shown to reprogram HCC cell glycolysis by directly targeting HK2 (hexokinase 2) in HCC^[36]^. miR-195 regulated cell cycle progression via targeting CDK6, CCNE1, CDC25A, and CDK4, leading to the abnormal cell proliferation in HCC development^[37]^. miR-122 is known to be a highly expressed miRNA and one of the most abundant liver specific miRNAs, accounting for about 70% and 52% of the whole hepatic total miRNAs in adult mice and humans, respectively^[38-40]^. miR-122 also plays a significant role in the regulation of cholesterol and fatty acid metabolic pathway in the liver, and reduction of miR-122 expression in the liver contributes to the development of steatohepatitis^[41-43]^. Tsai *et al*.^[44]^ reported that liver-specific miR-122 knockout mice appear normal but develop steatohepatitis for dysregulation of lipid metabolism. Importantly, knockdown of miR-122 in the liver also promoted development of liver fibrosis and carcinogenesis. In addition, Simerzin *et al*.^[45]^ characterized one mechanism by which reduction of miR-122 expression induces HCC, and revealed that miR-122 activated p53 by targeting Mdm2 (mouse double minute 2 homolog), known as the negative regulator of p53. Roy *et al*.^[46]^ reported integrated analysis of the miRNA and mRNA expression in the liver tissues from a mouse model of HCC and observed a reduction of miR-193a-5p in murine and human HCC cells and tissues, leading to increased levels of NUSAP1 (nucleolar- and spindle-associated protein), which regulates cell proliferation by controlling spindle assembly and genomic stability^[47]^. In fact, an miR-193a-5p mimic and knockdown of NUSAP1 in Huh7 cells suppressed HCC development and migration. miR-206 inhibits tumor growth in HCC by targeting CCND1, cMET, and CDK6, and delivery of miR-206 into the liver almost completely suppressed growth of HCC in an AKT/Ras and cMyc HCC mouse model^[48]^.

The Wnt/β-catenin signaling pathway plays an essential role in the active process of liver development and governs the proliferation and differentiation of hepatocytes. Abnormal activity and gene mutations of this pathway are known to be involved in HCC development^[49]^. miR-148b targeting WNT1 contributes to cell growth in HCC and functions as a tumor suppressor in HCC^[50]^. Another study suggested that downregulation of miR-766-3p promotes HCC cell progression by targeting the Wnt3/ Protein regulator of cytokinesis-1 pathway as a tumor suppressor gene in HCC^[51]^.

### miRNAs as oncogenes

**Figure 1:**
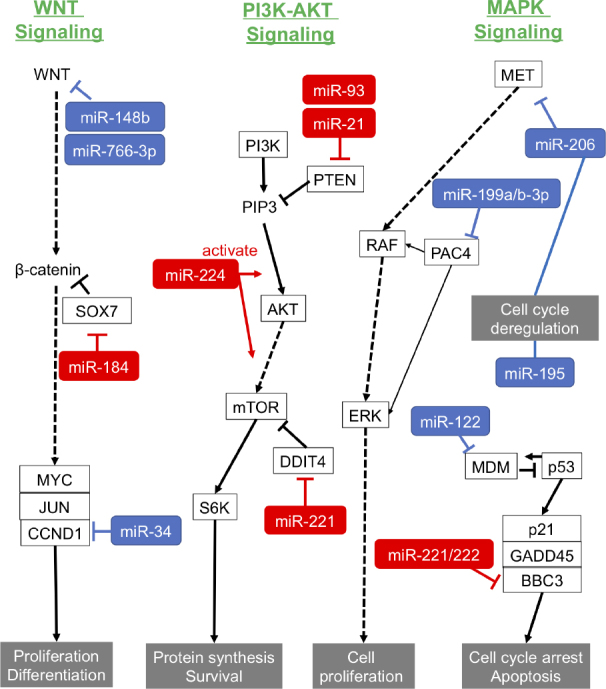
Roles of oncomiRs and suppressor miRNAs in carcinogenesis pathways of HCC. Red: oncomiRs; blue: suppressor miRNAs. PTEN: phosphatase and tensin homolog; oncomiRs: oncogenic miRNAs; miRNAs: microRNAs; HCC: hepatocellular carcinoma; PI3K: phosphoinositide 3-kinase; MAPK: mitogen-activated protein kinases; MET: mesenchymal-epithelial transition factor; PAC4: proteasome assembly chaperone 4 ; ERK: extracellular regulated kinase; MDM: mouse double minute; BBC3: Bcl-2-binding component 3; GADD45: growth arrest and DNA-damage-inducible 45 alpha; S6K: ribosomal protein S6 kinase; DDIT4: DNA-damage-inducible transcript 4; mTOR: mechanistic target of rapamycin; SOX7: SRY-box 7; CCND1: Cyclin D1

Recent studies have shown that some miRNAs, called oncogenic miRNAs (oncomiRs), regulate cell proliferation and the apoptotic processes that are important in HCC forming and growth [Table 2 and Figure 1].

**Table 2:** List of oncomiRs and suppressor miRNAs

OncomiRs	Target(s)	Ref.
miR-21	PTEN	[52]
	HBP1-p53-SREBP1C pathway	[53]
miR-93	PTEN, CDKN1A	[60]
miR-106b	DAB2 (disabled homolog 2)	[61]
miR-184	SOX7	[59]
miR-221	p53	[62]
	p27 and/or DDIT4	[56]
miR-221/222 cluster	BBC3	[57]
miR-224	AKT signaling	[58]
Tumor suppressor miRNAs		
miR-122	p53/MDM2	[45]
miR-148b	WNT1	[50]
miR-193a-5p	NUSAP1	[46]
miR-195	CDK6, CCNE1, CDC25A, and CDK4	[37]
miR-199a/b-3p	PAK4/Raf/MEK/ERK pathway	[35]
miR-199a-5p	HK2	[36]
miR-206	CCND1, cMET, and CDK6	[48]
miR-766-3p	Wnt3a/PRC1 pathway	[51]

miRNAs: microRNAs; PTEN: phosphatase and tensin homolog; MDM2: mouse double minute 2; BBC3: bcl-2 binding component 3; NUSAP1: nucleolar- and spindle-associated protein; HK2: hexokinase 2; CDKN1: Cyclin-Dependent Kinase Inhibitor 1; DAB2: Disabled homolog 2; SOX7: SRY-box 7; DDIT4: DNA-damage-inducible transcript 4; CDK6: Cyclin-dependent kinase 6; CCNE1: Cyclin E1; CDC25A: cell division cycle 25 homolog A: CDK4: cyclin-dependent kinase 4; PAK4: p21-Activated kinase 4; MEK: mitogen-activated protein kinase; ERK: extracellular regulated kinase; CCND1: cyclin D1; cMET: mesenchymal-epithelial transition factor; PRC1: protein regulator of cytokinesis-1

Phosphatase and tensin homolog (PTEN) is a tumor suppressor gene and is involved in the regulation of the cell cycle, preventing cells from growing and dividing rapidly. Excessive expression of miR-21 can promote HCC growth and spread by inhibiting PTEN expression^[52]^. Moreover, another study revealed that miR-21 contributes to hepatic steatosis and cancer progression by modulating p53 and Srebp1c pathway via target HBP1 (HMG-box transcription factor 1)^[53]^. miR-221 is over-expressed and associated with the tumor capsular infiltration that affects invasion and metastasis in HCC clinical samples, and miR-221 inhibits cell proliferation and apoptosis-mediating G1/S-phase arrest^[54]^. Pineau *et al*.^[55]^ showed that miR-221 overexpression promoted the growth of tumorigenic murine hepatic progenitor cells using a mouse HCC model, and that miR-221 could induce the tumor growth via suppressing p27 and/or DDIT4 (DNA damage-inducible transcript 4) expression. miR-222, which is in the miR-221/222 cluster and is upregulated in HCC tissues, promotes the progression of HCC by targeting the 3’UTR of the mRNA of BBC3 (Bcl-2 binding component 3)^[56]^.

Ma *et al*.^[57]^ showed that miR-224 acted as an oncogenetic regulator of HCC through the activation of AKT signaling^[58]^. miR-184 upregulated c-Myc and cyclin D1 and phosphorylated Rb protein by targeting SOX7, resulting in increasing proliferation in HCC cells^[59]^. As shown in Table 2, the targets of miR-93, miR-106b and miR-221 were PTEN, CDKN1A^[60]^, DAB2^[61]^ and p53^[62]^, respectively.

Recent findings suggest that miRNAs are involved in the abnormal epigenetic regulation of cancer cells^[63]^. In HCC, a recent study showed hypomethylation of a cancer-specific microRNA cluster in tumors from patients without HCV or HBV infection^[64]^. He *et al*.^[65]^ determined that oncomiRs, including miR-106b, miR-25, miR-93, miR-23a, and miR-27a, are hypomethylated and upregulated in HCC. Moreover, methylation of mature miRNAs may be related with carcinogenesis. Konno *et al*.^[66]^ showed that methylated miRNAs were increased in gastrointestinal cancer and methylated miR-200c-3p suppressed its target genes compared with unmethylated miR-200c-3p. In HCC, methylated miRNAs have not been reported yet, but it may be necessary to examine the relationship between methylation of miRNAs and tumors as well as expression of miRNAs.

### Involvement of miRNAs in HCC invasion and metastasis

Recent studies suggest the aberrant expression of miRNAs also affects the invasion and metastasis of HCC. miR-21, miR-34a, and miR-224 have been shown to be involved in proliferation, epithelial-mesenchymal transition (EMT), and metastasis of HCC^[67,68]^.

Zhou *et al*.^[69]^ reported that tumor-derived extracellular vesicles (EVs) miR-21 could convert hepatic stellate cells to cancer-associated fibroblasts by decreasing PTEN expression, leading to activation of the PDK1/AKT signaling pathway in HCC. In another study, miR-21 produced by endothelial progenitor cells led to cell migration, invasion, and EMT via induction of monocyte chemotactic protein 1 in liver cancer cells^[70]^. miR-224 inhibited Homeobox D10 directly and activated expression of p-PAK4 and MMP-9, which regulated remodeling, invasion, and metastasis in tumors^[71]^.

Extracellular vesicles are the endosome-derived vesicles with a diameter of 40-100 nm that are actively secreted by most cells, and they contain functional proteins, mRNAs, and miRNAs. It has been elucidated that HCC cell-secreted EVs can transfer miRNAs into recipient cancer cells, tumor microenvironment, and distant organs, thus promoting growth, migration, and invasion^[72,73]^. Lin *et al*.^[74]^ reported that EV-derived miR-210 secreted by HCC cells could be delivered into endothelial cells and directly suppressed SMAD4 and STAT6 expression, thereby promoting tumor angiogenesis. Hepatoma cell-secreted EV-derived miR-103 increases vascular permeability and enhanced tumor metastasis by inhibiting multiple endothelial junction proteins [VE-Cadherin (VE-Cad), p120-catenin (p120), and tight junction]^[75]^. Fang *et al*.^[76]^ showed that EV-derived miR-1247-3p secreted by highly metastatic HCC cells activates β1-integrin-NF-κB signaling in fibroblasts and converts into cancer-associated fibroblasts, resulting in the promotion of cancer metastasis.

### miRNAs and tumor immunity

In a recent study, immune checkpoint therapy targeting T cell-negative costimulatory molecules such as programmed cell death-1(PD-1) showed a therapeutic effect at a certain rate in many cancers. Emerging evidence suggests that tumor-derived microRNAs involve tumor immune escape by targeting immune checkpoint proteins and molecules^[77]^. Several miRNAs have been found to be downregulated in cancer cells to allow PD-L1 expression^[78,79]^.

In HCC, cancer-derived EVs also suppress CD8+ T-cell function by promoting Bregs, Tregs, or M2 macrophages^[80]^. HCC-derived miR-146a upregulated PD-1, TIGIT, and CTLA4 on T cells by promoting M2 tumor-associated macrophages and accelerated HCC progression^[81]^. Secreted EVs miR-23a from HCC cells under endoplasmic reticulum-stress conditions increased PD-L1 expression on macrophages, which then decreased expansion and function and promoted apoptosis in cytotoxic T lymphocyte^[82]^.

These miRNAs can be predictors of tumor response to treatment but also might be new therapeutic targets for HCC treatment. There is still a lack of research in this field, and further investigation of the relationship between tumor immunity and miRNAs is expected.

## Application of circulating miRNAs as clinical biomarkers

Early diagnosis is beneficial for the prognosis of cancer, including HCC. Serum alpha fetoprotein (AFP) is the most used biomarker for diagnosis of HCC but its sensitivity and specificity are only 60% and 90%, respectively^[83]^. Reliable and simple prognostic factors have not been established yet, but the identification of circulating miRNAs that are useful as biomarkers for HCC diagnosis, prognosis, and therapeutic response is expected^[84,85]^.

### Circulating miRNA as diagnostic biomarkers for HCC

Several reports have shown the potential of circulating miRNAs as a clinical biomarker in HCC diagnosis [Table 3].

**Table 3:** Circulating microRNAs as diagnostic biomarkers for HCC

Ref.	Body fluid	Case	Number	Control/Number	microRNAs	Regulation
Zhou *et al*.^[86]^	Plasma	HCC	457	NH167/CHB169/LC141	miR-21	UP
					miR-192	UP
					miR-801	UP
					miR-26a	DN
					miR-27a	DN
					miR-122	DN
					miR-223	DN
Jin *et al*.^[87]^	Plasma	HCC	116	NH79/CHB49/LC18	miR-1972	UP
					miR-193a-5p	UP
					miR-214-3p	UP
					miR-365a-3p	UP
Tan *et al*.^[88]^	Serum	HCC	261	NH173/LC233	miR-206	UP
					miR-141-3p	UP
					miR-433-3p	UP
					miR-1228-5p	UP
					miR-199a-5p	DN
					miR-122-5p	DN
					miR-192-5p	DN
					miR-26a-5p	DN
Zhang *et al*.^[89]^	Serum	HCC	115	NH40	miR-16-2-3p	UP
					miR-92a-3p	UP
					miR-107	UP
					miR-3126-5p	DN
Huang *et al*.^[90]^		HCC	3423	NH1887/CHB2403	miR-21	UP
					miR-122	UP
Yamamoto *et al*.^[91]^	Serum	HCC	353	NH1033/CH46/LC93	miR-320b	UP
					miR-663a	UP
					miR-4448	UP
					miR-4651	UP
					miR-4749-5p	UP
					miR-6724-5p	UP
					miR-6877-5p	UP
					miR-6885-5p	UP

HCC: hepatocellular carcinoma; NH: normal healthy; CHB: chronic hepatitis B; UP: upregulation; DN: downregulation; LC: liver cirrhosis

Zhou *et al*.^[86]^ investigated plasma microRNA expression in three independent cohorts, comprising 934 participants (healthy, chronic hepatitis B, cirrhosis, and HBV-related HCC). A microRNA panel (miR-122, miR-192, miR-21, miR-223, miR-26a, miR-27a, and miR-801) revealed high sensitivity of HCC diagnosis (AUC = 0.864 and 0.888 for the training and validation datasets, respectively). Jin *et al*.^[87]^ reported the plasma circulating miRNA profiling of 262 samples (116 HCC patients, 18 cirrhosis patients, 49 chronic hepatitis B, and 79 healthy controls) from three independent cohorts to find effective biomarkers that can distinguish the different liver disease stages of chronic hepatitis, cirrhosis, and HCC. They found four miRNAs (miR-1972, miR-193a-5p, miR-214-3p, and miR-365a-3p) that could distinguish HCC from non-HCC samples.

Tan *et al*.^[88]^ conducted a large-scale study of 667 subjects (261 HCC patients, 233 cirrhosis patients, and 173 healthy controls) to identify serum circulating miRNAs as reliable biomarkers in the diagnosis of HBV-related HCC. They identified an miRNA set (hsa-miR-206, hsa-miR-141-3p, hsa-miR-433-3p, hsa-miR-1228-5p, hsa-miR-199a-5p, hsa-miR-122-5p, hsa-miR-192-5p, and hsa-miR-26a-5p) that provided high diagnostic accuracy for HCC (healthy: AUC = 0.893; cirrhosis: AUC = 0.892). Zhang *et al*.^[89]^ also identified a miRNAs panel (miR-92-3p, miR-107, and miR-3126-5p) as a reliable diagnostic marker for HCC, especially for early stage patients (AUC = 0.975) and for low-level AFP HCC patients (AUC = 0.971). Huang *et al*.^[90]^ performed comprehensive analysis of large Chinese datasets in the Medline, Embase, and Chinese National Knowledge Infrastructure (MEDLINE) databases to elucidate effective miRNAs for the early diagnosis of HCC. miR-21 and miR-122 were significantly upregulated in multiple studies on HCC and capable of diagnosing HCC. However, since this result may be specific to ethnic groups, an analysis that integrates multiracial data will be required in the future. Recently, Yamamoto *et al*.^[91]^ reported that an eight-miRNA panel (miR-320b, miR-663a, miR-4448, miR-4651, miR-4749-5p, miR-6724-5p, miR-6877-5p, and miR-6885-5p) could accurately distinguish HCC patients from healthy (AUC = 1.00; sensitivity, 97.7%; and specificity, 98.4%) and chronic hepatitis/cirrhosis patients (AUC = 0.99; sensitivity, 97.7%; and specificity, 94.7%) in a study of 344 HCC patients. Importantly, compared with serum AFP, the eight-miRNA panel showed high sensibility (stage I, 98%) at the early stage of HCC. Furthermore, they have started the prospective analysis of the eight-miRNA panel for HCC screening.

Validating and quantifying of miRNA can be influenced by the type of blood sample, collection protocol, and isolation and detection methods. The lack of standardized detection protocols and data normalization makes it difficult to compare the results of different studies, therefore leading to inconsistent findings in screening of circulating miRNAs. Recently, in a comprehensive analysis of miRNA expression in serum and plasma, there are distinct differences in both the number and quantification of detected miRNAs between serum and plasma samples^[92]^. Thus, it is necessary to use standardized protocols, including for sample collection, RNA isolation, and the selection of a suitable internal control for normalization.

### Circulating miRNA as prognostic and recurrence markers for HCC

Xu *et al*.^[93]^ analyzed the correlation between serum miR-122 expression and prognosis in 122 HCC patients, and their results showed that HCC patients with high expression of serum miR-122 had significantly better overall survival rate than those with low miR-122 levels. Over-expression of pretreatment plasma miR-122 is also correlated with early refractoriness in HCC patients with transarterial chemoembolization (TACE) treatment^[94,95]^. Li *et al*.^[96]^ found that high levels of serum miR-221 expression was associated with tumor size, cirrhosis, and tumor stage in 46 HCC patients. Their results suggest that circulating miR-221 expression could be a prognostic marker. In a large screening study, Zhang *et al*.^[97]^ investigated the potential prognostic miRNAs using data from 54 relevant articles and 6464 patients and identified circulating miR-148a and miR-192 as reliable prognostic markers in HCC. Jin *et al*.^[87]^ also revealed that six miRNAs (miR-424-5p, miR-101-3p, miR-128, miR-139-5p, miR-382-5p, and miR-410) were significantly related with overall survival rate in 116 HCC patients and had potential to be good biomarkers for predicting prognosis.

Several studies identified circulating miRNAs as therapy predictive markers^[98]^. Chuma *et al*.^[99]^ reported that serum miR-1246 predicted early tumor recurrence after hepatic resection. Cho *et al*.^[100]^ revealed that the levels of circulating miR-26a and miR-29a before treatment were prognostic markers for liver transplantation-free survival in hepatitis B virus-related HCC patients. Serum levels of miR-181a-5p and miR-122 were found to have potential to predict response of HCC to sorafenib^[101,102]^. Recently. Teufel *et al*.^[103]^ reported some plasma miRNAs associated with response to regorafenib treatment in HCC.

### Extracellular vesicle-derived miRNAs in diagnosis

miRNAs are more stable in serum exosomes because the lipid bilayer protects them from degradation by RNases^[104]^. Therefore, blood samples are appropriate for the evaluation of extracellular vesicle-derived miRNAs as potential biomarkers in HCC diagnosis and prognosis. Moreover, EVs can specifically reflect their original cell types and conditions and may be able to detect early stages of cancer as special biomarkers^[105-107]^.

In their recent studies, Sohn *et al*.^[108]^ isolated EVs from the serum of CHB, cirrhosis, and HCC patients and found over-expression of several EV-derived miRNAs, namely miR-18a, miR-221, miR-222, and miR-224, and downregulation of serum EV-derived miR-101, miR-106b, miR-122, and miR-195 in HCC patients. Additionally, they compared expression patterns of EV-derived miRNAs with those of serum circulating miRNAs. They revealed that EV-derived miRNAs were more sensitive to distinguishing HCC from CHB/cirrhosis than serum circulating miRNAs. Other studies found high levels of EV-derived miR-21, an abundant miRNA expressed in HCC tissue, and this was an independent predictor of mortality and disease progression^[109-111]^. Mjelle *et al*.^[112]^ analyzed paired microRNAs profiling of tumor and normal tissue and circulating EVs from HCC patients using small RNA sequencing methods. There was a consistent positive correlation of miR-21 expression between circulating EVs and HCC tissue.

To identify specific microRNAs in exosomes from the sera of patients with recurrent HCC, Sugimachi *et al*.^[113]^ performed miRNA analyses of samples from 59 patients who underwent living donor liver transplantation. They found significantly lower levels of miR-718 in the serum EVs of HCC cases with recurrence. Downregulation of miR-718 increased expression of *HOXB8*, a known oncogene, resulting in carcinogenesis and metastasis. Another study showed serum EV-derived miR-122 and miR-21 could be predictive biomarkers in HCC patients with liver cirrhosis treated with TACE^[114]^. The research revealed that patients with a higher miR-122 ratio (after TACE/before TACE) had significantly longer disease-specific survival, compared to patients with a lower miR-122 ratio.

As mentioned above, although several studies have investigated the clinical significance of EV-derived miRNAs in HCC, there are still few reports on serum EV-derived microRNA in HCC. A larger cohort of HCC should be included and further improvements in the isolation and detection of serum EV-derived miRNA are expected.

Recently, lncRNAs and proteins in exosomes have also been the focus of research to understand the pathogenesis of cancer and its progression. Yuan *et al*.^[115]^ showed that lncRNA-ATB acted as a key regulator of TGF-β signaling pathways and induced EMT and tumor cell invasion in HCC. Interestingly, Hoshino *et al*.^[116]^ found EVs from different tumor types bear integrins that target these exosomes to specific organs and trigger signaling pathways, thereby initiating pre-metastatic niche formation. They suggested that the integrin contained in exosomes could be used as a biomarker to predict metastasis destination in cancer patients. The research of exosomes in HCC will contribute to gain new biological insights and provide novel diagnostics and therapeutics targets.

## miRNA-based therapeutics in HCC

miRNAs are also considered as potential therapeutic targets for cancer. miRNA therapeutics are based on targeting oncomiRs or mimicking tumor suppressor miRNAs^[117]^. Double-stranded miRNA mimics aim to upregulate tumor suppressor miRNA, whereas anti-oncomiRs, short interfering RNA (siRNA), and single stranded anti-sense oligonucleotides are designed to specifically bind to and inhibit miRNAs that bear oncogenic properties.

Since there are abundant ribonucleases, administered miRNA is rapidly degraded in blood. One solution to this is chemical modification, e.g., using phosphorothioate-containing oligonucleotides, 2-*O*-methyl-(2-*O*-Me)/2-*O*-methoxyethyl oligonucleotides (2-*O*-MOE), 2-fluoro oligonucleotides (2’-F), and locked nucleic acid (LNA) oligonucleotides^[118]^. In liver disease, miravirsen, an LNA-containing anti-miR-122 that inhibits translation of HCV genome, entered phase II clinical trials in 2017, and the result of the clinical trial was positive^[119-122]^. N-acetylgalactosamine (GalNAc) platform enables specific, targeted uptake into hepatocytes. GalNAc-conjugated anti-miR targeting miR-103 and miR-107 that regulate insulin sensitivity is being used in patients with non-alcoholic fatty liver disease and type 2 diabetes in a Phase I/II trial^[123,124]^.

Another solution for miRNA-based therapeutics is a drug delivery system. There are two types of miRNA delivery systems, viral and non-viral^[125]^. Viral vector systems usually use retroviruses, lentiviruses, and adenoviruses or adeno-associated viruses. Viral vectors have higher transfection efficiency but are more toxic and immunogenic. For this reason, safer non-viral carriers are being developed for clinical applications. Non-viral vectors include lipid-based nanocarriers, polymeric vectors/dendrimer-based vectors, and cell-derived membrane vesicles. A liposome-encapsulated miR-34a mimic (MRX34) was able to block HCC growth in more than a third of the treated animals^[126]^. Furthermore, MRX34 was used for patients with various tumors, including HCC (*n* = 14) in a Phase I trial. One patient with HCC achieved a prolonged PR lasting 48 weeks, and four patients experienced SD lasting more than four cycles^[127]^. Sato *et al*.^[128]^ developed a multifunctional envelope nanodevice (MEND) incorporating various functions, including polyethylene glycol modification and introduction of membrane-permeable peptides. A pH-sensitive lipid YSK05 (YSK05-MEND) specifically delivered higher amounts of the anti-miR-122 to the liver^[129]^. Recently, extracellular vesicles are considered to play important roles in intercellular communication and are also used as effective drug carriers^[130-132]^. EVs have low cytotoxicity and antigenicity. Application studies on nanocarriers that encapsulate miRNA, siRNA, *etc*. inside extracellular vesicles by ultrasonic irradiation or electroporation and deliver them to target cells are being performed^[133]^. In a recent study, EVs were isolated from human plasma and engineered with miR-31 and miR-451a. The engineered EVs promoted apoptosis of HCC *in vitro*^[134]^. Moreover, in another study, EVs were isolated from human adipose tissue-derived mesenchymal stromal cells (ASC) modified with a lentiviral vector expressing miR-125b. The miR-125b containing EVs inhibited proliferation in HCC cells by suppressing p53 expression^[135]^.

## Conclusion

**Figure 2:**
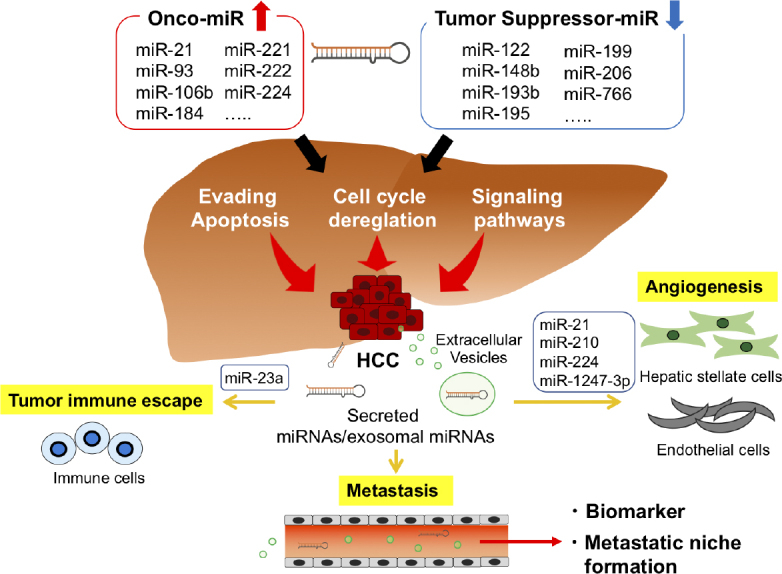
Summary of miRNAs involved in the development and progression of HCC. HCC: hepatocellular carcinoma

miRNAs are being considered as new biomarkers and potential therapeutic targets for HCC [Figure 2]. To date, many miRNAs have been identified as regulators of target genes involved in carcinogenesis, invasion, and metastasis in HCC. In addition, circulating EV-derived miRNAs secreted by cancer cells may become strong cancer biomarkers and novel therapeutic targets, and miRNA-based therapies have evolved and are being developed to be safer and more effective. Further research is needed to determine the prospects of new miRNA-based anticancer therapies for HCC treatment. However, several issues need to be resolved to adopt circulating miRNAs for diagnostic application in clinical routine. Normalization is one of the most controversial issues. The choice of a reference gene can have a significant impact on measuring the level of transcripts and, thus, on the biological interpretation of the data. In addition, standardization of miRNA processing is required, from sample collection and sample storage to RNA isolation and reverse-transcription. If these current limitations can be overcome, miRNAs may eventually be implemented in diagnostic algorithms, as well as be used to predict the clinical outcomes of HCC patients.

## Declarations

### Copyright

© The Author(s) 2020.
